# Chicken CH25H inhibits ALV-J replication by promoting cellular autophagy

**DOI:** 10.3389/fimmu.2023.1093289

**Published:** 2023-02-15

**Authors:** Tingting Xie, Min Feng, Xi Zhang, Xiaoqi Li, Guodong Mo, Meiqing Shi, Xiquan Zhang

**Affiliations:** ^1^Guangdong Provincial Key Laboratory of Agro-animal Genomics and Molecular Breeding, College of Animal Science, South China Agricultural University, Guangzhou, China; ^2^Key Lab of Chicken Genetics, Breeding and Reproduction, Ministry of Agriculture, Guangzhou, Guangdong, China; ^3^State Key Laboratory for Conservation and Utilization of Subtropical Agro-Bioresources, South China Agricultural University, Guangzhou, Guangdong, China; ^4^Division of Immunology, Virginia-Maryland Regional College of Veterinary Medicine, University of Maryland, College Park, MD, United States

**Keywords:** chicken, ALV-J, *CH25H*, ISG, autophagy

## Abstract

Autophagy plays an important role in host antiviral defense. The avian leukosis virus subgroup J (ALV-J) has been shown to inhibit autophagy while promoting viral replication. The underlying autophagic mechanisms, however, are unknown. Cholesterol 25-hydroxylase (CH25H) is a conserved interferon-stimulated gene, which converts cholesterol to a soluble antiviral factor, 25-hydroxycholesterol (25HC). In this study, we further investigated the autophagic mechanism of CH25H resistance to ALV-J in chicken embryonic fibroblast cell lines (DF1). Our results found that overexpression of *CH25H* and treatment with 25HC promoted the autophagic markers microtubule-associated protein 1 light chain 3 II (LC3II) and autophagy-related gene 5(ATG5), while decreased autophagy substrate p62/SQSTM1 (p62) expression in ALV-J infection DF-1 cells. Induction of cellular autophagy also reduces the levels of ALV-J gp85 and p27. ALV-J infection, on the other hand, suppresses autophagic marker protein LC3II expression. These findings suggest that CH25H-induced autophagy is a host defense mechanism that aids in ALV-J replication inhibition. In particular, CH25H interacts with CHMP4B and inhibits ALV-J infection in DF-1 cells by promoting autophagy, revealing a novel mechanism by which CH25H inhibits ALV-J infection. Although the underlying mechanisms are not completely understood, CH25H and 25HC are the first to show inhibiting ALV-J infection *via* autophagy.

## 1 Introduction

Avian leukosis virus subgroup J (ALV-J) belongs to the retroviridae family, is a genus of retroviruses, and causes secondary diseases such as chicken tumor disease and immunosuppression (1). It is well known that ALV-J infection causes enormous economic loss for the global poultry industries, including reduced egg production, stunted growth, and increased mortality. Unfortunately, vaccines or treatments to prevent ALV-J infection do not exist yet. The current strategy for eradicating ALV-J is to eliminate positive chickens. In contrast, the host’s strategy of resisting the ALV-J virus can save a lot of workforces and material resources and ensure the diversity of chicken breeds.

Host cells use pattern recognition receptors (PRRs) to detect pathogen-associated molecular patterns (PAMPs) during viral infection. Subsequently, antiviral responses are initiated. Interferon-mediated antiviral responses can be considered the main manifestation of innate immunity, followed by inducing hundreds of interferon-stimulated genes (ISGs) (2). Increasing studies support that ISG products exerts its antiviral function in innate immunity by regulating and manipulating autophagy (3). RNA-dependent eIF2α protein kinase (PKR) was demonstrated essential for HSV-1 autophagic degradation (4). During early viral infections, ribonuclease L (RNase L), another ISG product, activates autophagy to inhibit viral multiplication (5). Autophagy is an evolutionarily conserved process for lysosome self-digestion of harmful components, aberrant cytosolic constituents, and intracellular pathogens (6). During viral infection, autophagy recognizes viral components and plays a key role in restricting viral replication (7). Thus, autophagy has also been shown to play an important role in antiviral defense. However, with a long-term struggle between viruses and host cells, viruses have evolved diverse strategies to evade or exploit autophagy for their survival (8–10). These data indicate that autophagy is considered a double-edged sword in antiviral immunity (11). Previous studies have shown that ALV-J negatively regulates autophagy through the GADD45β/MEKK4/P38MAPK signaling pathway, then mediates apoptosis and promotes viral replication (12, 13). Interestingly, *GADD45β* is an anti-ALV-J gene in chickens (14). Thus, we speculate that ALV-J evades the host immune response by inhibiting host cellular autophagy.

Cholesterol 25-hydroxylase (*CH25H*), a multitransmembrane endoplasmic reticulum (ER)-associated enzyme, mainly function is to catalyze excess cholesterol to produce 25-Hydroxycholesterol (25HC); meanwhile, *CH25H* is a classic antiviral ISG, promotes resistance against a variety of enveloped viruses, including SARS-CoV, MERS-CoV and SARS-CoV-2 Ebola virus (EBOV), human immunodeficiency virus (HIV), Zika virus (ZIKV), Rift Valley fever virus (RVFV), Nipah virus (NiV) and ALV-J (15–17). However, HSV-1 UL41 escapes the antiviral function of *CH25H* through its endonuclease activity (18). Previously study have reported that *CH25H* broadly inhibits virus entry by 25HC; As a soluble oxysterol, 25HC inhibits virus entry by blocking membrane fusion between cells and viruses (15). Increasing studies proposed that CH25H and 25-Hydrxycholesterol (25HC) broadly resist viral infection through multiple mechanisms, such as manipulating cholesterol metabolism to inhibit viral adsorption entry and release, or inhibition of viral replication through interactions with viral components, modulating inflammation, innate and adaptive immunity, that may not involve the production of 25HC to inhibit viral replication (19).

Therefore, we further investigated whether CH25H may regulate autophagy to exert antiviral function in the current study. Through exogenous *CH25H* overexpression and 25HC treatment, we find that *CH25H* and 25HC could promote autophagy in DF-1 cells. It is further demonstrated that CH25H interacts with CHMP4B and inhibits ALV-J infection. Our results demonstrate a novel mechanism of CH25H resistance to ALV-J and advance our understanding of host innate antiviral immunity to ALV-J infection.

## 2 Materials and methods

### 2.1 Cell culture and virus infection

DF-1 cells were purchased from the American Type Culture Collection (ATCC, Manassas, VA, USA). DF-1 cells were cultured in Dulbecco’s modified Eagle’s medium (DMEM) (Gibco, USA) supplemented with 10% FBS and maintained at 37°C and 5% CO_2_.

The ALV-J strain, SCAU-HN06, was a kind gift from Professor Ming Liao and Professor Weisheng Cao (College of Veterinary Medicine, South China Agricultural University). Briefly, DF-1 cells were infected with ALV-J train SCAU-HN06 (10^5^ TCID_50_/0.1mL). After 2 h incubation, washed by 1×PBS, the media was replaced with DMEM and added 1% FBS. For certain experiments, cells were selected with a suitable inducer concentration for 2 h prior to ALV-J virus infection.

### 2.2 Construction of overexpression plasmids, siRNA, and cell transfection

Flag-tagged *CH25H* plasmid was stored in our laboratory (17). The *CHMP4B*, *TSG101*, *RPL23*, *PCBP2* were cloned into a PCAGGS vector with a C-terminal myc tag. PCR amplification primer sequences are as follows: chicken *CHMP4B* forward primer, 5’-ATGTCGGGGATCCTGGG-3’ and reverse primer, 5’-TTACATGTTTCCTGCCCAAG-3’; and chicken *RPL23* forward primer, 5’-CGCCTCCGTCTCTTCCG-3’ and reverse primer 5’-TGCACAAAGATGAGCACGTT-3’. The vector of chicken *TSG101* and chicken *PCBP2* was synthesized by Wuhan Genecreate Biological Engineering Co., Ltd (Wuhan, China). Small interfering RNA (siRNA) targeting Chicken *TSG101*, *CHMP4B*, *RPL23*, *PCBP2*, and normal control (NC) siRNA (siN0000001-1-5) were synthesized by RiboBio (RiboBio Inc, China). The siRNA sequences are as follows:

Chicken TSG101-siRNA: CCTATCTCATGTGCTATCA (dTdT)-3’;
Chicken CHMP4B-siRNA: 5’- AGCCAAGAAGAAAGAGGAA (dTdT)-3’;
Chicken RPL23-siRNA: 5’-CGGAAATCGTACAGAAGAA (dTdT)-3’;
Chicken PCBP2-siRNA: 5’- TTTGCAGGCGGTCAGCTGA (dTdT)-3’.

DF-1 cells were transfected with these plasmids or siRNAs using Lipofectamine 3000 reagent, according to the manufacturer’s instructions.

### 2.3 Reagents and antibodies

25HC was purchased from Sigma-Aldrich. Caspase-1 activity assay kit was purchased from Beyotime Institute of Biotechnology. Lipofectamine 3000 was purchased from Invitrogen. Annexin V-FITC and PI apoptosis detection kits were purchased from Keygen Biotech Company (Nanjing, China). Antibodies used in the present study were as follows: ALV-J envelope protein JE9-specific mouse monoclonal antibody (provided by Professor Qin Aijian of Yangzhou University), rabbit anti-LC3II antibody (NB100-2220SS, Novus Biologicals), rabbit anti-ATG5 antibody (12994, Cellsignal), mouse monoclonal anti-flag antibody (AP007M, Bioworld), rabbit anti- SQSTM1/p62 antibody (P0067, Sigma-Aldrich), and anti-myc antibody (A02060, Abbkine), rabbit anti-β-actin antibody (AF5003, Beyotime), goat anti-rabbit IgG (H+L) antibody (A21020, Abbkine), Goat anti-mouse IgG/HRP antibody (A25112, Abbkine).

### 2.4 Reverse transcription-quantitative PCR

Total RNA was extracted from DF-1 cells at the indicated time points using TRIzol reagent (TaKaRa, Kusatsu, Japan) and reversely transcribed to cDNA using a PrimeScript RT Reagent kit (TaKaRa, Kusatsu, Japan). qPCR analysis was performed on a CFX96 PCR system (Bio-Rad) using SYBR green fast mixture (Bio-Rad, Hercules, CA, USA). Expression levels were quantified using the 2 ^−ΔΔC(T)^ method and normalized to GAPDH. Primers used for RT-qPCR are listed in **Supplementary Table 1**.

### 2.5 Caspase-1 activity assays and ALV-J p27 detection

Caspase-1 activity assay kits (C1102, Beyotime Biotechnology, Shanghai, China) were used to analyze Caspase-1 activities, following the manufacturer’s instructions. According to a previous study, the expression levels of ALV group-specific antigen (p27) were analyzed (17).

### 2.6 Flow cytometry

Apoptosis were analysed by double staining with Annexin V-FITC (fluorescein isothiocyanate) and propidium iodide (PI). Annexin V binds to phosphatidylserine on the outer plasma membrane of apoptotic cells, while PI labels necrotic cells with membrane damage. The prepared cells were gently washed three times with pre-chilled PBS. Subsequently, 500 μL Annexin V binding buffer was added, and the cells were mixed gently. Cell suspensions were then incubated with 5 μL FITC Annexin V and 5 μL propidium iodide (PI) for 15 min at room temperature in the dark. Then, the percentage of viable cells (Annexin V-/PI-), early apoptotic cells (Annexin V+/PI-), and late apoptotic or necrotic cells (Annexin V+/PI+) were analyzed by flow cytometry (BD AccuriC6, Biosciences, USA), and data was processed using FlowJo7.6 software.

### 2.7 Confocal microscopy

DF-1 cells were cultured in 24-well PET cell culture inserts. Flag-tagged CH25H plasmid and control plasmid were transfected into DF-1 cells. After 24 h, DF-1 cells were fixed with 4% paraformaldehyde, permeabilized with 2% Triton-X-100, blocked with blocking solution (P0252, Beyotime) for 20 min, and incubated with primary antibody anti-Flag overnight at 4°C. Secondary antibody FITC-labeled Goat Anti-Mouse (A0568, Beyotime) was added, and the nuclei were stained with DAPI (P0131, Beyotime). Imaging was performed with a Leica TCS-SP5 confocal microscope with a ×40 objective.

### 2.8 Transmission electron microscopy

The DF-1 cells in a six-well culture plate were divided into five treatment groups: DF-1 cells without any treatment (NC group), DF-1 cells infected with ALV-J train SCAU-HN06 (10^5^ TCID_50_/0.1mL) (ALV-J group), DF-1 cells transfected with the plasmid pflag-EGFP following by ALV-J (10^5^ TCID_50_/0.1mL) infection (EGFP group), DF-1 cells transfected with the plasmid pflag-CH25H following by ALV-J (10^5^ TCID_50_/0.1mL) infection (CH25H group), and DF-1 cells pretreated with 1 μM 25HC for 12 h (17) following by ALV-J (10^5^ TCID_50_/0.1mL) infection (25HC group). Cells were harvested at 48 h after ALV-J infection and were fixed in 2.5% glutaraldehyde (Solarbio, China) at 4°C for 6 h. Resin impregnation, adding neat resin and polymerizing at 65°C. The intracellular structure of the stained cells was observed with a transmission electron microscope (TEM) (Hitachi HT7800).

### 2.9 Western blot analysis

After treatment, total protein was extracted from DF-1 cells and quantified by the Bradford BSA Protein Assay Kit (Beijing Dingguo Changsheng Biotechnology, China). Proteins were separated by SDS-polyacrylamide gel electrophoresis (4-15% SDS-PAGE) and were wet-transferred onto PVDF membrane (Bio-Rad) for immunoblotting. Membranes were incubated with the primary antibody (1:1000) at 4°C overnight. Protein bands were visualized with an Odyssey FC infrared imaging system and analyzed with Image Studio (LICOR Biosciences, NE, USA).

### 2.10 Immunoprecipitation- mass spectrometry analysis

Transfected DF-1 cells were washed three times with pre-cooled PBS, lysed with IP lysate buffer, and extracted with total protein buffer (Thermo Fisher Scientific), supplemented with 2% PMSF (ST507, Beyotime), then centrifuge at 13,000 rpm for 10 minutes at 4°C. 50 μL Protein G magnetic beads (Thermo Fisher Scientific) were conjugated with 5 µg anti-Flag and were gently mixed on a rotator mixer at 4°C for 2 h. Subsequently, the pretreated supernatants were further incubated with anti-Flag antibody-conjugated magnetic beads on a rotator mixer overnight at 4°C. Finally, the bound proteins were eluted for SDS‐PAGE. The proteins pulled‐down by IP were subjected to mass spectrometry analysis performed by Shenzhen Wininnovate Bio Co., Ltd (Shenzhen, China).

### 2.11 Yeast two-hybrid assay

According to the signal peptide and transmembrane analysis, we constructed pBT3-STE-CH25H as a bait vector and pPR3-N-CHMP4B as a prey vector, while combinations of pTSU2-APP and pNubG-Fe65 were used as positive controls, combinations of pTSU2-APP and pPR3-N were used as negative controls. Combinations of pBT3-STE-CH25H and pPR3-N, pBT3-STE-CH25H and pPR3-N-CHMP4B, pBT3-STE-CH25H and POST-NubaI, pTSU2-APP and pNubG-Fe65, pTSU2-APP and PPR3-N were co-transformed into the yeast strain NMY51. Different co-transformed yeast cells were carried out ten-fold serial dilution, and 4 ten-fold series dilutions of each co-transformant were dropped synthetic dropout (SD) medium lacking Leu and Trp and SD medium lacking Ade, His, Leu, and Trp with 30 mm 3AT. Photographs were taken after 5 d of incubation.

### 2.12 Statistical analysis

All experiments were independently repeated at least twice, and each experiment included three replicates. Statistical analysis and graph generation were performed using GraphPad Prism 5 (GraphPad Software Inc., USA). All statistical data are presented as the mean ± SEM, and significance was assessed using two-way ANOVA. Differences with *P*-values < 0.05 (*), 0.01 (**), and 0.001 (***) were statistically significant.

## 3 Results

### 3.1 ALV-J infection induces apoptosis in DF-1 cells

**Figure 1:**
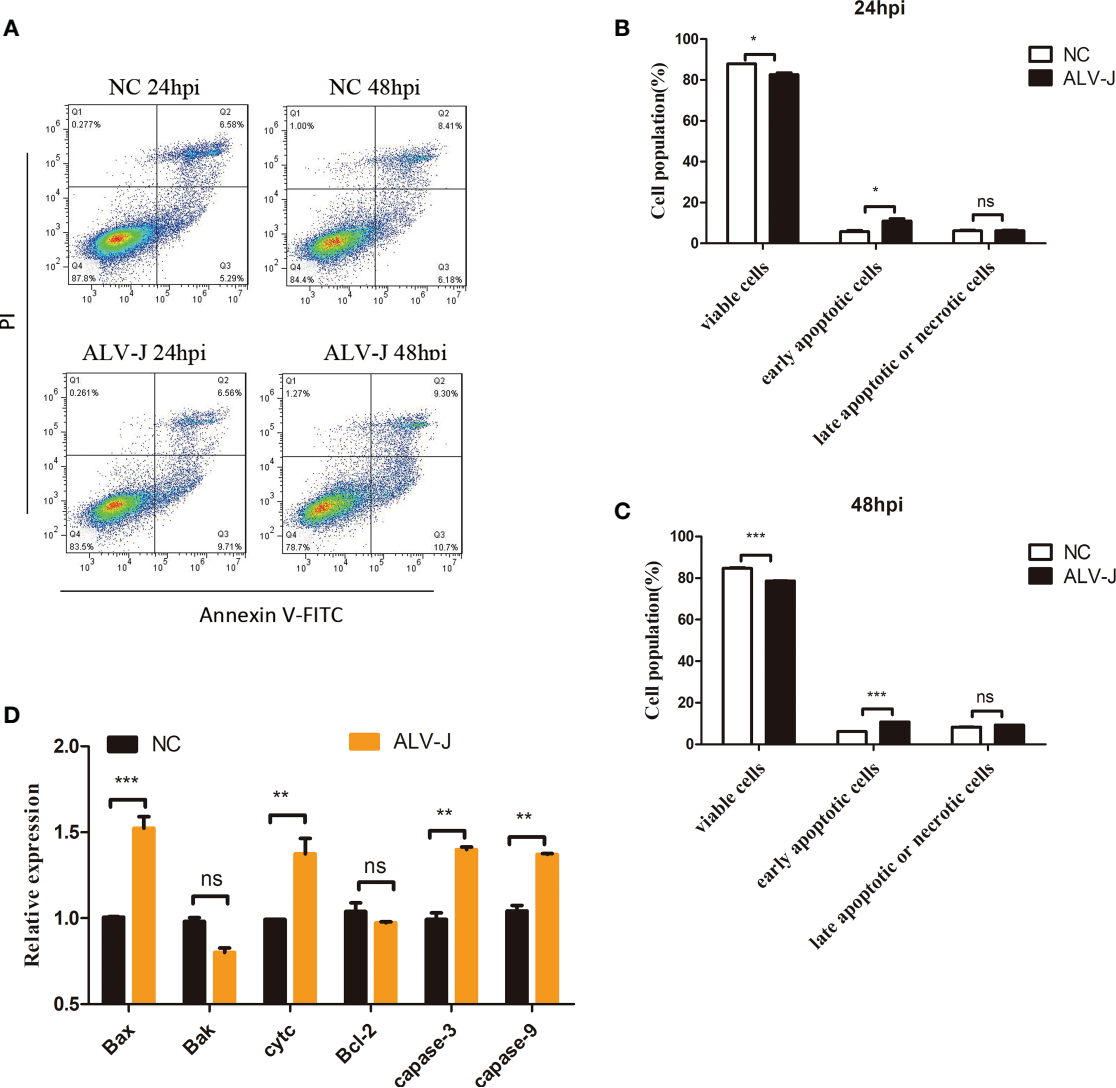
ALV-J infection induces DF-1 cells apoptosis. **(A)** DF-1 cells were infected with ALV-J (10^5^ TCID_50_/0.1mL) for the indicated time. Normal cells (NC) with no infection as a negative control. Apoptosis of DF-1 cells was measured by flow cytometry. **(B, C)** Statistical analysis of viable cells, early apoptotic cells, late apoptotic cells or necrotic cells rate. **(D)** qRT-PCR analysis of apoptosis-related genes (*Bax*, *Cyt c*, *Caspase-3* and *Caspase-9*, *Bak*, and *Bcl-2)* in ALV-J infected cells. hpi represents hours post infection. Data shown are the means ± SEM of at least two independent experiments. *P* values were calculated using unpaired Student’ t-test. A *P*-values < 0.05 were considered statistically significant differences, and a *P*-values < 0.01 were considered highly statistically significant differences. *P < 0.05, **P < 0.01, ***P < 0.001, ns: Non-significant.

Apoptosis is the most common form of programmed cell death (20). Apoptosis was measured in DF-1 cells infected with ALV-J to identify whether ALV-J infection induces activation of apoptosis. Flow cytometry results showed that DF-1 cells undergo apoptosis at 24 h or 48 h of ALV-J infection (**Figures 1A-C**). Moreover, compared to normal control (NC) cells, the mRNA expression of apoptosis-related genes including *Bax*, *Cyt c*, *Caspase-3*, and *Caspase-9* were significantly upregulated in DF-1 cells infected with ALV-J, while *Bak*, and *Bcl-2* were not significantly different (**Figure 1D**). These results suggested that ALV-J infection induced DF-1 cells apoptosis.

### 3.2 *CH25H* inhibited early apoptosis and increased late apoptosis or necrosis

Programmed cell death (PCD) or regulated cell death (RCD) plays an important role in cancers as well as in diseases (21). Our previous study had proven that expression levels of the *CH25H* could be significantly induced in the immune system organs of clinical chickens and ALV-J infected cells (17, 22), which suggested that the host may exerts an antiviral function by inducing the expression of *CH25H*. To further explore the ability of *CH25H* to resist ALV-J virus, DF-1 cells were transfected with pflag-*CH25H* for 24 h prior to being infected with ALV-J. The result of qRT-PCR (**Supplementary Figure 1A**), ELISA (**Supplementary Figure 1B**) and immunoblot (**Supplementary Figure 1C**) analyses showed that overexpression of *CH25H* can inhibit the viral titer of 10^5^ TCID_50_/0.1mL ALV-J. While CH25H exerts its anti-ALV-J function, it also induces host cell death (**Supplementary Figure 1D**), which suggested that CH25H may be closely related to cell apoptosis. Thus, we hypothesized that *CH25H* restricts ALV-J infection by regulating host apoptosis.

**Figure 2:**
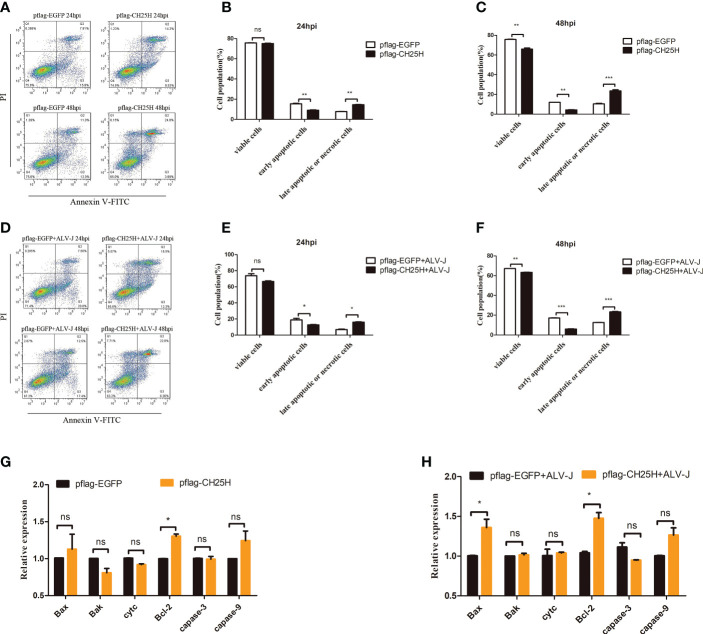
CH25H induces activation of programmed cell death. **(A)** DF-1 cells were transfected with pflag-CH25H and control plasmid pflag-EGFP for the indicated time, cell apoptosis was detected after AnnexinV FITC/PI labeling by flow cytometry, respectively. **(B, C)** Statistical analysis of viable cells, early apoptotic cells, late apoptotic cells or necrotic cells rate. **(D)** DF-1 cells were transfected with pflag-CH25H or pflag-EGFP for 24 h and infected with ALV-J (10^5^ TCID_50_/0.1mL), cell apoptosis was detected after AnnexinV FITC/PI labeling by flow cytometry at 24 and 48 h infection. **(E, F)** Statistical analysis of viable cells, early apoptotic cells, late apoptotic cells or necrotic cells rate. **(G)** DF-1 cells were transfected with pflag-CH25H and control plasmid pflag-EGFP for 48 h, qRT-PCR analysis of the mRNA expression of apoptosis-related genes (*Bax*, *Cyt c*, *Caspase-3*, *Caspase-9*, *Bak*, *Bcl-2* and *Bcl-xl*). **(H)** DF-1 cells that were transfected with pflag-CH25H or pflag-EGFP were infected with ALV-J at 24 h. At 48 hpi, qRT-PCR analysis of the mRNA expression of apoptosis-related genes (*Bax*, *Cyt c*, *Caspase-3*, *Caspase-9*, *Bak*, and *Bcl-2*). hpi represents hours post infection. Data shown are the means ± SEM of at least two independent experiments. *P* values were calculated using unpaired Student’ *t*-test. A *P*-values < 0.05 were considered statistically significant differences, and a *P*-values < 0.01 were considered highly statistically significant differences. *P < 0.05, **P < 0.01, ***P < 0.001, ns: Non-significant.

DF-1 cells overexpressing *CH25H* were detected by flow cytometry to test this hypothesis. Flow cytometry results showed that overexpression of *CH25H* significantly inhibited early apoptosis, but the percentage of late apoptotic or necrotic cells were significantly increased (**Figures 2A-C**). And,we found that overexpressing *CH25H* inhibited early apoptosis response in ALV-J infected DF-1 cells, while late apoptotic or necrotic cells were significantly increased (**Figures 2D-F**). Next, key apoptosis-related genes including *Bax*, *Cyt c*, *Caspase-3*, *Caspase-9*, *Bak*, and *Bcl-2* were examined. Notably, the genes encoding the proapoptotic factors, *Bax*, *Bak*, *Cyt c*, *Caspase-3*, and *Caspase-9*, were not significantly different, while the anti-apoptotic marker *Bcl-2* was significantly up-regulated in DF-1 cells overexpressing *CH25H*, as compared to vector control (**Figure 2G**). Besides, DF-1 cells were transfected with *CH25H* expression plasmid followed by ALV-J infection, and those encoding the proapoptotic factors, *Bak*, *Cyt c*, *Caspase-3*, and *Caspase-9*, were not significantly different, while the anti-apoptotic marker *Bcl-2* was significantly up-regulated, as compared to ALV-J infected vector control (**Figure 2H**). Therefore, overexpression of *CH25H* inhibited apoptosis and increased late apoptosis or necrosis, and *CH25H* did not exert its anti-ALV-J effect through apoptosis.

Pyroptosis is an inflammatory cell death dependent upon the inflammatory caspases that plays a crucial role in defense against microbial infections (21, 23). In order to explore whether CH25H restricts ALV-J infection by regulating pyroptosis, we then examined the expression levels of caspase-1 and inflammatory factors *IL-1β*, *IL-18*, *NLRP*3. As a result, we found that *CH25H* overexpression elevates levels of *IL-1β*, *IL-18* and *NLRP3* but not *caspase-1* (**Supplementary Figure 2**), suggesting that no pyroptosis occurred. These results suggested that overexpression of *CH25H* does not induce pyroptosis.

### 3.3 Both the overexpression of *CH25H* and the treatment with 25HC promote autophagy

Autophagy is considered a mechanism of programmed cell death and is an evolutionarily conserved self-eating phenomenon and has multiple effects on antiviral innate immunity (24–26). A previous study showed that ALV-J inhibits autophagy and promotes viral self-replication (12). We therefore further hypothesized that CH25H inhibits ALV-J by promoting autophagy. We overexpressed *CH25H* in DF-1 cells to verify this hypothesis, and then autophagy-related proteins were analyzed by immunoblot. According to the fact that rapamycin (Rapa) is an autophagy inducer, a concentration of 1 μM Rapa (13) was used to analyze the expression levels of microtubule-associated protein 1 light chain 3 II (LC3II), autophagy-related gene 5(ATG5), and SQSTM1/p62 proteins (p62). Detection of LC3 protein levels by immunoblotting is one of the most common methods to measure autophagy, and comparing LC3II levels between samples is an appropriate method to determine autophagy (27). ATG5, an essential factor for proper autophagosome formation and maturation (28), was significantly increased. p62, which is a substrate of autophagy, was significantly downregulated.

**Figure 3:**
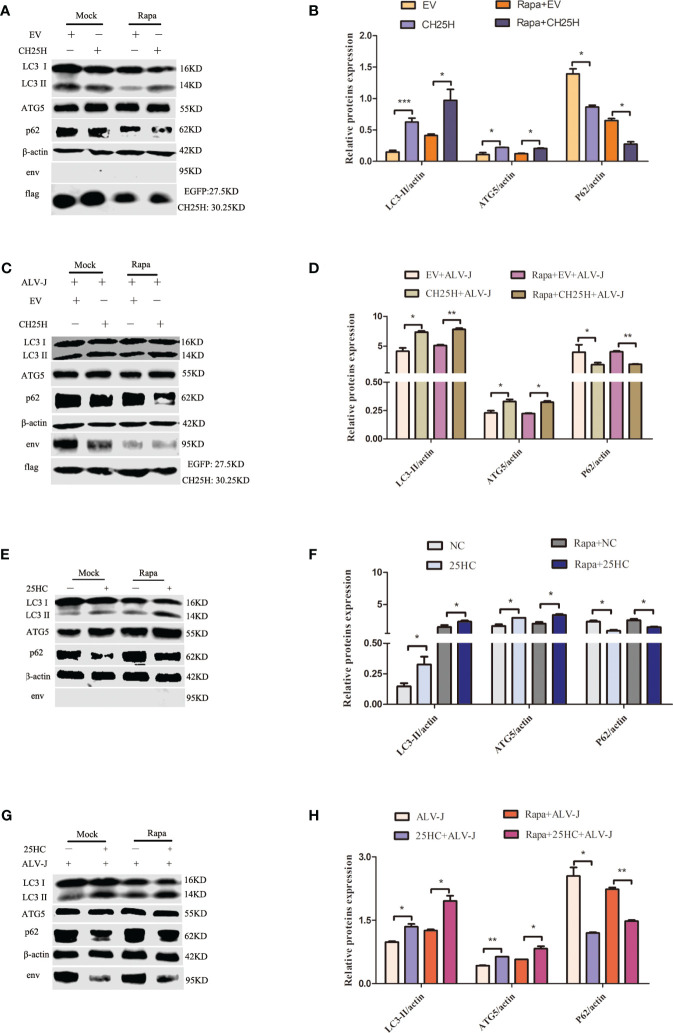
Both the overexpression of CH25H and the treatment with 25HC promote autophagy. **(A)** DF-1 cells were pretreated with 1 μM Rapa for 2 h and then were transfected with pflag-CH25H or pflag-EGFP for 48 h. Expression levels of LC3II, ATG5, p62, ALV-J env, flag-tagged CH25H, and flag-tagged EGFP were analyzed by immunoblot. **(B)** Quantification of LC3II/β-actin, ATG5/β-actin, and p62/β-actin were analyzed by Image Studio software. **(C)** DF-1 cells that were pretreated with 1 μM Rapa for 2 h were transfected with pflag-CH25H or pflag-EGFP for 24 h, prior to being infected with ALV-J (10^5^ TCID_50_/0.1mL). The expression levels of LC3II, ATG5, p62, ALV-J env, flag-tagged CH25H, and flag-tagged EGFP were analyzed by immunoblot. **(D)** Image Studio software was used to quantify LC3II/β-actin, ATG5/β-actin, and p62/β-actin. **(E)** DF-1 cells were pretreated with 1 μM 25HC for 12 h and replaced with fresh medium for 48 h, and the expression levels of LC3II, ATG5, p62, and ALV-J env were assessed by immunoblot. **(F)** Quantification of LC3II/β-actin, ATG5/β-actin, and p62/β-actin were analyzed by Image Studio software. **(G)** DF-1 cells were pretreated with 25HC at a concentration of 1 μM for 12 h, followed by infection with ALV-J (10^5^ TCID_50_/0.1mL) for 48 h. The expression levels of LC3II, ATG5, p62, and ALV-J env were assessed by immunoblot. **(H)** Image Studio software was used to quantify LC3II/β-actin, ATG5/β-actin, and p62/β-actin by Image Studio software. Data shown are the means ± SEM of at least two independent experiments. *P* values were calculated using unpaired Student’ *t*-test. A *P* values < 0.05 were considered statistically significant differences, and a *P* < 0.01 were considered highly statistically significant differences. *P < 0.05, **P < 0.01, ***P < 0.001.

As expected, *CH25H* overexpression significantly increased the LC3II level compared with the empty vector (**Figures 3A, B**). In addition, immunoblot analysis showed that the levels of LC3II and ATG5 were significantly promoted, while p62 was significantly decreased, at 48 h post-infection (hpi), with overexpressing *CH25H* before ALV-J virus infection (**Figures 3C, D**).

DF-1 cells were pretreated with 25HC to explore whether 25HC induces autophagy, and the immunoblot assays for LC3II, ATG5, and p62 were performed to detect autophagy. The results showed that the expression levels of LC3II and ATG5 were significantly promoted, while p62 were significantly decreased, compared with normal control (NC) cells (**Figures 3E, F**). Meanwhile, in ALV-J-infected DF-1cells, 25HC pretreatment significantly increased the expression levels of LC3II and ATG5, but significantly inhibited the expression level of p62 (**Figures 3G, H**). Altogether, these results indicated that both the overexpression of *CH25H* and the treatment with 25HC promoted autophagy.

### 3.4 CH25H inhibits ALV-J by promoting autophagy

**Figure 4:**
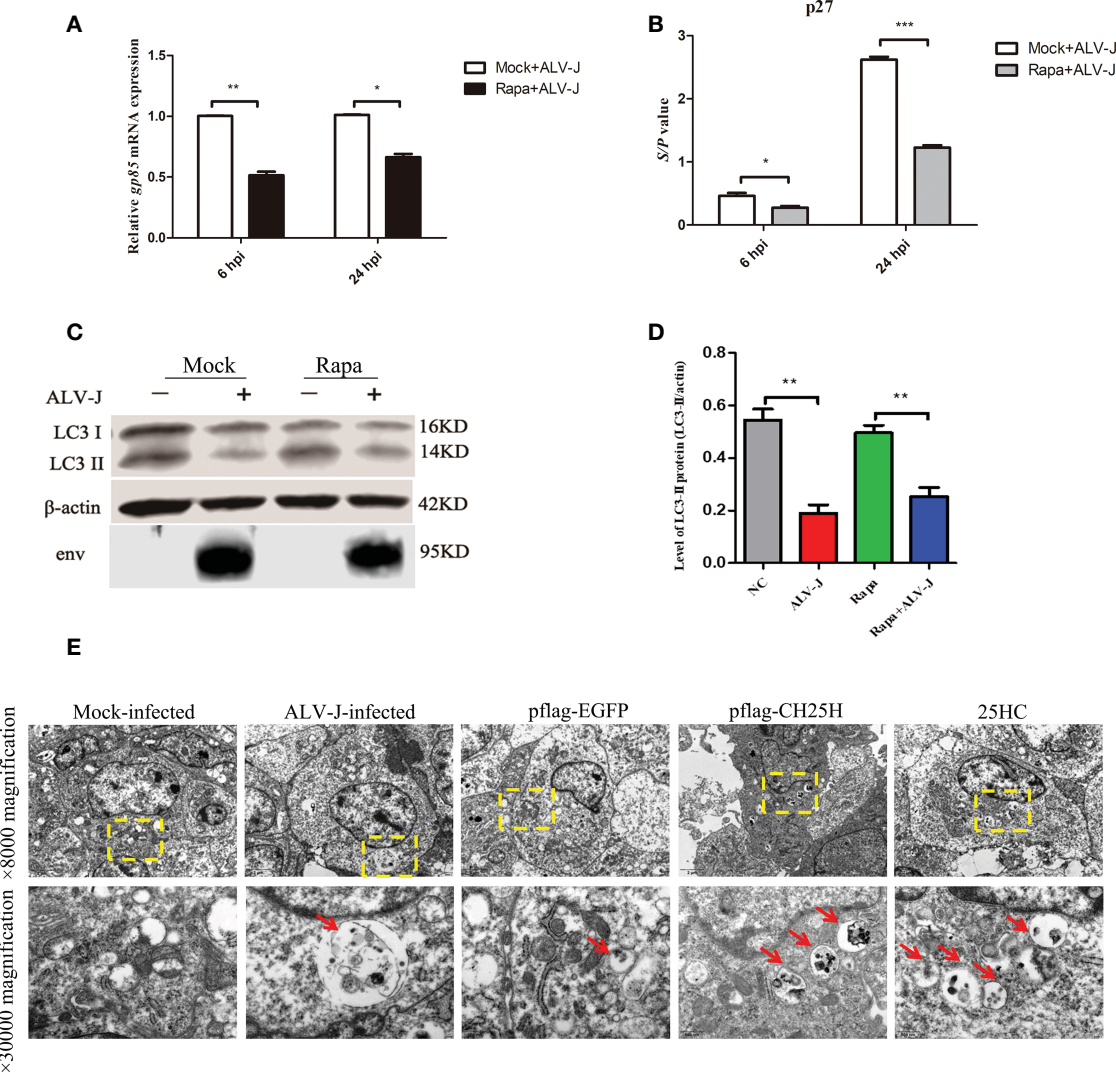
CH25H inhibits ALV-J by promoting autophagy. **(A)** DF-1 cells were pretreated with Rapa for 12 h and then infected with ALV-J (10^5^ TCID_50_/0.1mL) for 6 h and 24 h, respectively. The gp85 mRNA levels were detected by qRT-PCR for the indicated time. **(B)** The ALV-J p27 protein was measured by ELISA for the indicated time. **(C)** DF-1 cells were pretreated with 1 μM Rapa for 2 h and then infected with ALV-J (10^5^ TCID_50_/0.1mL) for 24 h. Expression levels of LC3II were analyzed using immunoblot. **(D)** The quantification of LC3II/β-actin was analyzed by Image Studio software. **(E)** Autophagosomes were observed with TEM. CH25H transfected and 25HC treatment cells showed autophagosomes, and few autophagic vacuoles were observed for ALV-J infected and controlled vector-transfected cells. The red arrow indicates an autophagosome. The top row pictures are ×8000 magnification. The bottom row pictures are ×30000 magnified from the yellow box of the top row. hpi represents hours post infection. Data shown are the means ± SEM of at least two independent experiments. *P* values were calculated using unpaired Student’ *t*-test. A *P*-values < 0.05 were considered statistically significant differences, and a *P*-values < 0.01 were considered highly statistically significant differences. *P < 0.05, **P < 0.01, ***P < 0.001.

Based on the above results, we speculated that CH25H-induced autophagy acts as a defense mechanism to inhibit ALV-J replication. To test this hypothesis, DF-1 cells were pretreated with Rapa for 12 h prior to being infected with ALV-J for 6 and 24 h. qRT-PCR and ELISA analyses showed that the expression level of ALV-J gp85 (**Figure 4A**) and ALV-J p27 (**Figure 4B**) were significantly decreased in Rapa-treated cells compared to Rapa-untreated (mock) cells. At the same time, autophagy is also detected by immunoblot analysis with an anti-LC3B antibody. As expected, ALV-J infection inhibits autophagy (**Figures 4C, D**). Next, autophagosomes were further determined by TEM. Consistently, autophagosomes were present in both CH25H transfected and 25HC treated cells, while a comparative few autophagosomes were observed for ALV-J-infected and control vector-transfected cells (**Figure 4E**). Altogether, these data suggested that CH25H-induced autophagy is a host defense mechanism to inhibit ALV-J viruses.

### 3.5 CH25H binds to CHMP4B and inhibits ALV-J infection by promoting autophagy

**Figure 5:**
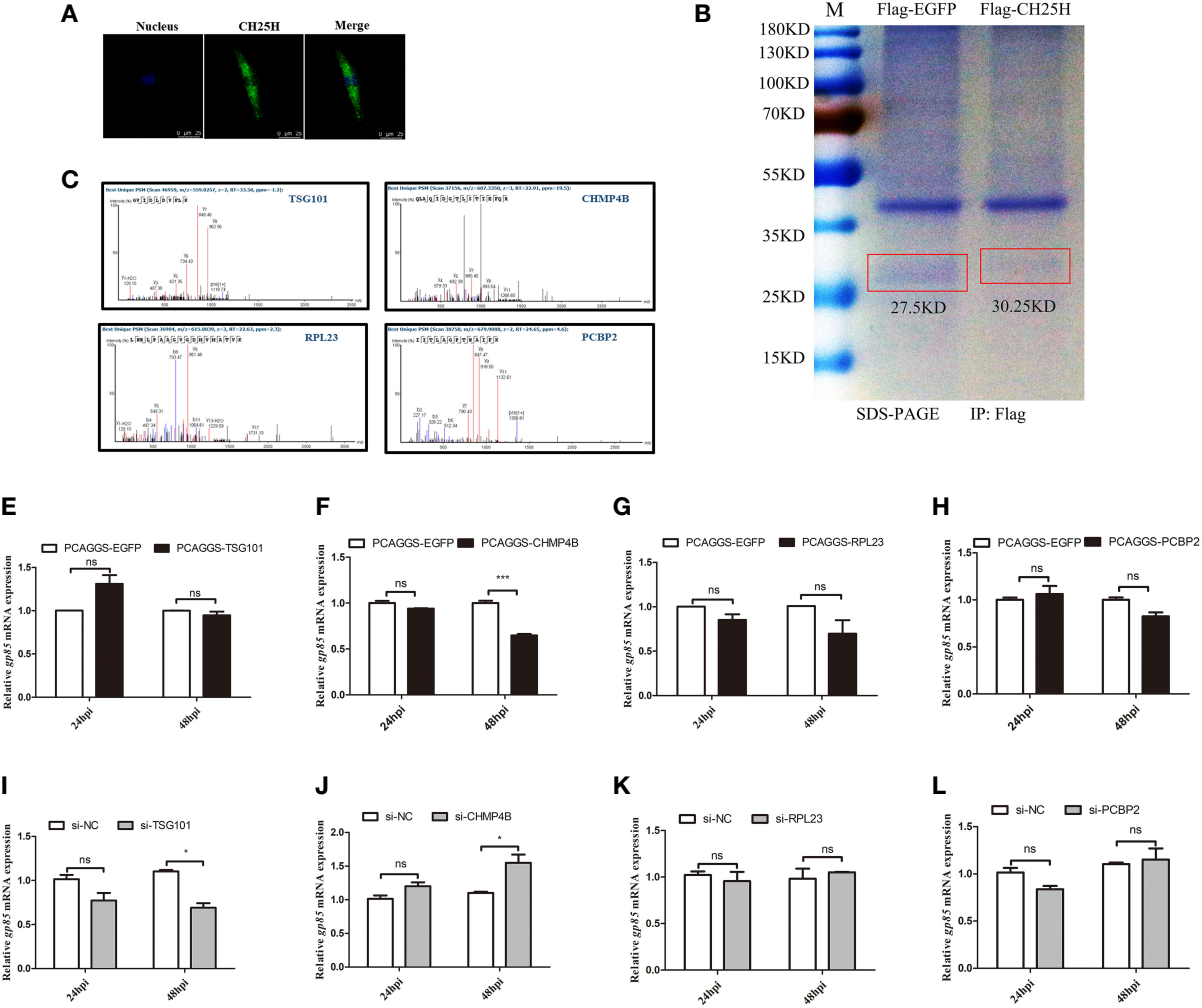
The identification of candidate CH25H-interacting proteins. **(A)** flag-tagged CH25H was expressed in DF-1 cells for 24 h and subsequently infected with ALV-J (10^5^ TCID_50_/0.1mL) for 48 h. CH25H localization was analysed by immunofluorescence with anti-FLAG antibody (green). Nuclei were visualized with DAPI (blue). Scale bar, 75 μm. **(B)** The Coomassie brilliant blue staining of *CH25H* detected with an anti-FLAG antibody. **(C)** Mass spectrogram of candidate CH25H-interacting proteins, including TSG101, CHMP4B, RPL23, and PCBP2. **(D-G)** *TSG101* **(D)**, *CHMP4B* **(E)**, *RPL23* **(F)**, and *PCBP2* **(G)** were overexpressed in DF-1cells for 24 h and subsequently infected with ALV-J (10^5^ TCID_50_/0.1mL), respectively. gp85 mRNA levels were detected by qRT-PCR at 24 hpi and 48 hpi. **(H-K)** Small interfering RNA (siRNA) was used to silence *TSG101* **(H)**, *CHMP4B* **(I)**, *RPL23* **(J)**, and *PCBP2* **(K)** DF-1 cells for 24 h and subsequently infected with ALV-J (10^5^ TCID_50_/0.1mL), respectively. gp85 mRNA levels were detected by qRT-PCR at 24 hpi and 48 hpi. *P < 0.05, ***P < 0.001, ns: Non-significant.

A separate study proposed that CH25H likely acts through cholesterol-independent mechanisms that inhibit viral replication (19). Thus, we hypothesized that CH25H inhibits ALV-J replication through interacting with proteins in the autophagy pathway. To prove this hypothesis, we performed IP-MS analysis and a two-hybrid assay to identify CH25H-interacting proteins. The flag-tagged CH25H expression plasmid and the control plasmid were transfected into DF-1 cells for 24 h and infected with the ALV-J virus. Confocal microscopy analysis showed that exogenous CH25H proteins were located in the cytoplasm in DF-1 cells (**Figure 5A**), which prompted us to consider that CH25H-interacting proteins are localized in the cytoplasm. Subsequently, we screened for possible CH25H target proteins by Co-IP (**Figure 5B**) combined with IP-MS experiments. About 65 unique proteins were identified in CH25H overexpression cells followed by ALV-J infection, compared with the EGFP overexpression control group (**Supplementary Table 2**), in which 10 interacting proteins were localized to the cytoplasm (**Supplementary Figure 3A**). Referring to the candidate proteins function annotation, candidate proteins ontology classification, candidate proteins biological process analysis, molecular functions analysis, and KEGG pathway analysis shown in **Supplementary Figures 3B-D** and **Supplementary Table 2**, 4 proteins (**Figure 5C**) were expected as candidate CH25H-interacting proteins.

The overexpression vectors of selected candidate proteins were overexpressed in DF-1 cells followed by ALV-J infection. The overexpression efficiencies were shown as **Supplementary Figures 4A-D**. qRT-PCR analyses results showed that overexpression of TSG101 had not significant different effect on ALV-J gp85 expression (**Figure 5D**); however, ALV-J gp85 was significantly decreased in cells overexpressing CHMP4B at 48 h (**Figure 5E**) but were not significantly different in cells overexpressing of RPL23 (**Figure 5F**) and PCBP2 (**Figure 5G**), compared to empty vector control. Conversely, silencing of TSG101 inhibits the expression of ALV-J gp85 at 48 hpi (**Figure 5H**), and silencing of CHMP4B promotes the expression of ALV-J gp85 at 48 hpi (**Figure 5I**); silencing of RPL23 (**Figure 5J**) and PCBP2 (**Figure 5K**) had not significant different effect on ALV-J gp85 expression. The interference efficiencies were shown as Supplementary (**Figure 4E–H**). Therefore, we speculated that CH25H might interact with CHMP4B and inhibited ALVJ replication.

**Figure 6:**
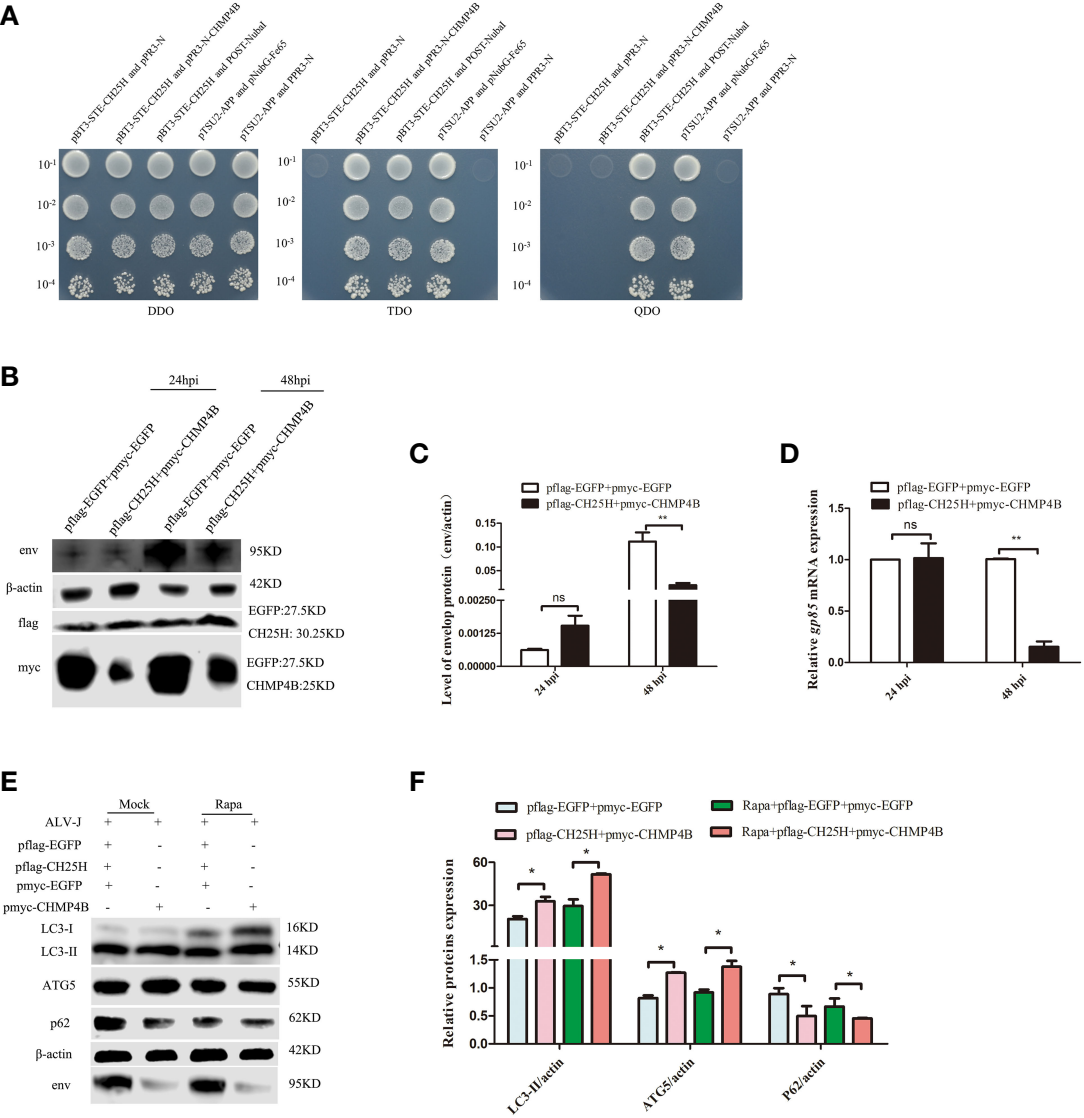
CH25H binds to CHMP4B and inhibits ALV-J replication by promoting autophagy. **(A)** A direct interaction between CH25H and CHMP4B was suggested from Yeast two-hybrid assay. **(B-F)** DF-1 cells were co-transfected with flag-tagged CH25H and myc-tagged CHMP4B plasmids for 24 h, subsequently infected with ALV-J (10^5^ TCID_50_/0.1mL). The expression levels ALV-J env, flag-tagged CH25H, and myc-tagged CHMP4B were assessed by immunoblot **(B)**. Relative expression levels of ALV-J env were quantified based on protein expression levels of β-actin **(C)**. Relative mRNA levels of ALV-J gp85 were detected by qRT-PCR at the indicated time **(D)**. **(E)** The expression levels of LC3II, ATG5, p62, and ALV-J env were analyzed by immunoblot. **(F)** Quantification of LC3II/β-actin, ATG5/β-actin, and p62/β-actin were analyzed by Image Studio software. EGFP represents the control. hpi represents hours post infection. Data shown are the means ± SEM of at least two independent experiments. *P* values were calculated using unpaired Student’ *t*-test. A *P*-values < 0.05 were considered statistically significant differences, and a *P*-values < 0.01 were considered highly statistically significant differences. *P < 0.05; **P < 0.01, ns: Non-significant differences.

Next, the interaction between CH25H and CHMP4B was verified by yeast two-hybrid assays. As expected, yeast two-hybrid assay results showed that CH25H interacted with CHMP4B (**Figure 6A**). Moreover, co-transfection of *CH25H* and *CHMP4B* significantly decreased the protein expression level of ALV-J env (**Figures 6B, C**), and significantly reduced the mRNA level of ALV-J gp85 (**Figure 6D**), at 48 hpi. Finally, whether co-overexpressing of *CH25H* and *CHMP4B* affected autophagy was investigated. The results clearly demonstrated that LC3II and ATG5 protein levels were significantly increased, whereas p62 expression levels were significantly decreased, in CH25H and CHMP4B co-overexpressing DF-1 cells infected with ALV-J. Altogether, the above data support that CH25H binds to CHMP4B and inhibits ALV-J replication by promoting autophagy.

## 4 Discussion

*CH25H* is an important ISG against viral infection. Our previous study had proven that *CH25H* could be induced by ALV-J and IFN-α and inhibits ALV-J infection through the production of 25HC (17). Another previous study suggested that ALV-J infection reduces autophagy during viral replication in DF-1 cells (12), which were consistent with the results from our current study (**Figures 4C, D**). Therefore, there might be certain crosstalk between *CH25H* restricting ALV-J infection and autophagy. We speculated that *CH25H* might regulate autophagy to exert antiviral function. Here, we have demonstrated that CH25H and 25HC promoted autophagy to inhibit ALV-J infection. ALV-J infection induces apoptosis and inhibits autophagy in DF-1 cells. Conversely, induction of cellular autophagy inhibits ALV-J infection. In addition, we identified a novel anti-ALV-J mechanism, which CH25H is binding to CHMP4B and promote autophagy to inhibit ALV-J infection (**Figure 6**).

The programs of cell death includes apoptosis, autophagy, pyroptosis, necrosis, and oncosis (29–31). Apoptosis and autophagy are considered two important processes for host intracellular immunity against infectious pathogens (32). Emerging evidence indicates that apoptosis may play a role in the host’s defense against viral infection (33). Our previous study showed that ACSL1, which is known to have antiviral ALV-J function, promotes host cells apoptosis (34). We therefore think that apoptosis is most likely the host’s immune response against ALV-J infection. This study showed that ALV-J infection induces apoptosis in DF-1 cells. However, cells overexpressed *CH25H* and then infected with ALV-J inhibited early apoptosis and induced late apoptosis or or necrosis, in which apoptosis-related proteins, *Caspase-3* and *Caspase-9* (35), were not significantly upregulated. *Bax* or *Bak* is required for caspase activation and is considered a key channel in the intrinsic pathway of apoptosis (36). Likewise, *Bax* and *Bak* were not significantly upregulated in CH25H-overexpressed cells. But the antiapoptotic gene *Bcl-2* was significantly upregulated in CH25H-overexpressed cells. These results suggested that *CH25H* did not exert its anti-ALV-J effect through apoptosis. And *CH25H* inhibits early apoptosis is to be further explored in the future.

Pyroptosis has been implicated in eliminating pathogenic infections (37). However, we demonstrated that *CH25H* overexpression elevates levels of *IL-1β*, *IL-18* and *NLRP3* but not Caspase-1 activity (**Supplementary Figure 3**). *IL-1β* promotes the inflammatory response and causes leakage of immune cells, and *IL-18* is known to stimulate local inflammatory responses (25). *NLRP3* inflammasome plays a pivotal role in protecting the host from infection (38).

Furthermore, we were surprised to find that CH25H and 25HC promoted autophagy during ALV-J infection. Our previous study confirmed that CH25H inhibited ALV-J infection by catalyzing cholesterol to 25HC (17). There is growing evidence that autophagy affects IFN-I responses by regulating the expression of IFN-I and IFNAR receptor (39–42). Likewise, IFN-I and ISG products can utilize autophagy to regulate antiviral immunity (43–45). *PKR*, a classical antiviral ISG, degrades HSV-1 viruses through autophagy (4). The IFN-β-inducible protein SCOTIN prevents HCV infection by mediating autophagy, degrading HCV nonstructural 5A (NS5A) protein (46). RNase L initiates antiviral initiation through autophagy-mediated activation of JNK and PKR (47). This study reveals a novel mechanism of CH25H against ALV-J infection, in which CH25H interacts with CHMP4B to inhibit ALV-J infection in DF-1 cells (**Figures 6A-D**). Similarly, co-transfection of CH25H and CHMP4B promotes cellular autophagy (**Figures 6E, F**). Autophagy is a mechanism of cellular self-degradation. Cells degrade their substances, including proteins and organelles, which require the assistance of the endosomal sorting complex required for transport (ESCRT) (48). ESCRTS are required for regulating division from receptor sorting in endosomes to cell membrane division and budding of enveloped viruses, vesicle formation at endosomes, cytokinesis (49). CHMP4B is a subunit of the endosomal sorting complex (ESCRT-III) (50). Studies have reported that CC2D1A protein binds to CHMP4B protein, thereby regulating CHMP4B and inhibiting the budding of HIV virions (51). In the research on progeria syndrome, autophagy relies on the ESCRT-III subunit pathway to regulate nuclear membrane deformation induced by premature aging (52). In the future, whether CHMP4B competitively binds CH25H and thus affects the production of 25HC, needs extensive experimental verification; a more comprehensive mechanisms of how CH25H and CHMP4B regulate autophagy would be further explored.

**Figure 7:**
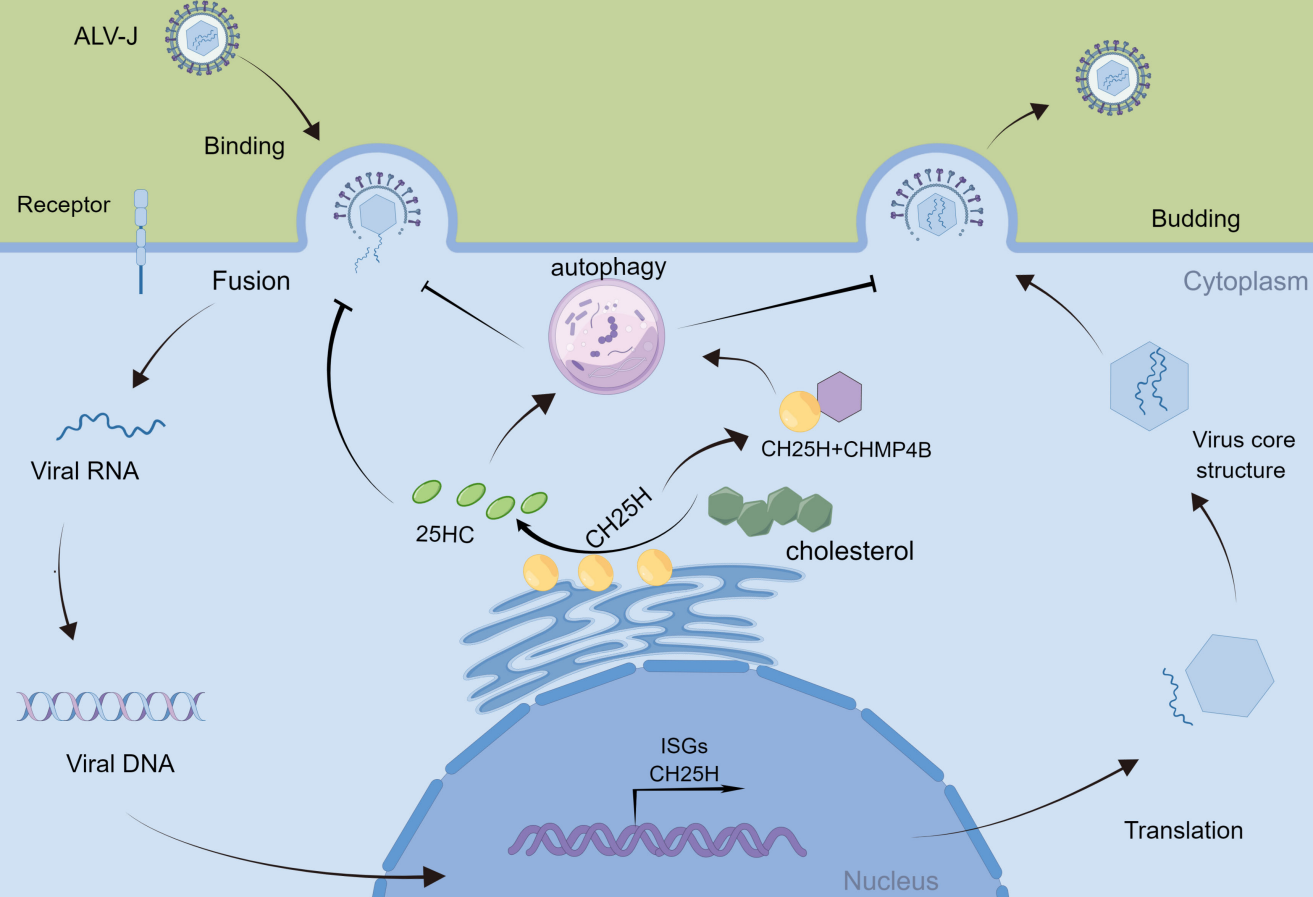
Schematic diagram of *CH25H* regulating ALV-J replication. ALV-J infection induces the production of Cholesterol 25-hydroxylase (*CH25H*). CH25H catalyzes the enzymatic oxidation of cholesterol to 25-hydroxycholesterol (25HC). CH25H and 25HC promote cellular autophagy, which in turn inhibits ALV-J. In addition, CH25H inhibits ALV-J by interacting with CHMP4B and promoting autophagy.

In conclusion, we have demonstrated that CH25H and 25HC promote autophagy to inhibit ALV-J infection; ALV-J infection suppresses cellular autophagy; in addition, CH25H interacts with CHMP4B and inhibits ALV-J infection in DF-1 cells by promoting autophagy (**Figure 7**). It provides new insight into immune response strategies between the host and ALV-J.

## Data availability statement

The original contributions presented in the study are included in the article/**Supplementary Material**. Further inquiries can be directed to the corresponding author.

## Author contributions

TX performed the experiments, collected and analyzed data, and contributed to the writing of the manuscript. MF provided advice and revised the manuscript. XiZ, XL and GM assisted in PCR amplification. XiqZ participated in the design and revised the manuscript. All authors read and approved the final manuscript.

## Supplementary material

Supplementary Figure 1 CH25H induces host cell death. DF-1 cells were transfected with flag-tagged CH25H for 24 h, subsequently infected with different virus titres of ALV-J for 48 h, respectively (10^5^, 10^4^, 10^3^, 10^2^ TCID_50_/0.1mL). **(A)** Relative mRNA levels of ALV-J gp85 were detected by qRT-PCR. **(B)** The ALV-J p27 protein was detected by ELISA at 24 hpi and 48 hpi. **(C)** The levels of ALV-J envelope (env) protein were assessed by Western blotting using the specific antibody JE9. **(D)** The growth of cells were observed under microscope. M represents standard protein markers, CH25H represents the overexpression of Chicken CH25H in DF-1 cells, EGFP represents the control plasmid. Data are shown as the means ± SEM of at least two independent experiments. P values were calculated using unpaired Student’ t-test. Differences with P-values < 0.05 were considered statistically significant differences, and P-values < 0.01 were considered highly statistically significant differences. *** P < 0.001.

Supplementary Figure 2 CH25H did not induce host cells pyroptosis. DF-1 cells were transfected with flag-tagged CH25H for 24 h, subsequently infected with ALV-J for 48 h, respectively (10^5^ TCID_50_/0.1mL). **(A)** A standard curves of pNA. **(B)** Caspase-1 activity was assayed using a caspase-1 activity assay kit. **(C)** Relative mRNA levels of *IL-1β*, *IL-18* and, *NLRP3* were detected by qRT-PCR.

Supplementary Figure 3 Bioinformatics analysis of candidate CH25H-interacting proteins. **(A)** Subcellular localization of candidate CH25H-interacting proteins as annotated by theUniProt database. **(B)** Biological process analysis of candidate CH25H-interacting proteins. **(C)** Molecular funtion of candidate CH25H-interacting proteins. **(D)** Pathway analysis of candidate CH25H-interacting proteins.

Supplementary Figure 4 The efficiency of overexpression and interference. *TSG101* **(A)**, *CHMP4B* **(B)**, *RPL23* **(C)**, and *PCBP2* **(D)** were overexpressed in DF-1cells for 24 h and subsequently infected with ALV-J (10^5^ TCID_50_/0.1mL), respectively. The efficiency of overexpression was examined by qRT-PCR. Similarly, specific siRNAs were used to silence *TSG101* **(H)**, *CHMP4B* **(I)**, *RPL23* **(J)**, and *PCBP2* **(K)** DF-1cells for 24 h and subsequently infected with ALV-J (10^5^ TCID_50_/0.1mL), respectively. The efficiency of interference was examined by qRT-PCR.

Supplementary Table 1 Primers used for qRT-PCR

Supplementary Table 2 The function annotation, ontology classification, biological process analysis, molecular functions analysis, and KEGG pathway analysis of candidate CH25H-interacting proteins.
