# Supplementary figures and images for: Chicken CH25H inhibits ALV-J replication by promoting cellular autophagy
Source: Front Immunol. 2023 Feb 15;14:1093289. doi: 10.3389/fimmu.2023.1093289 (PMC9975585; doi:10.3389/fimmu.2023.1093289)

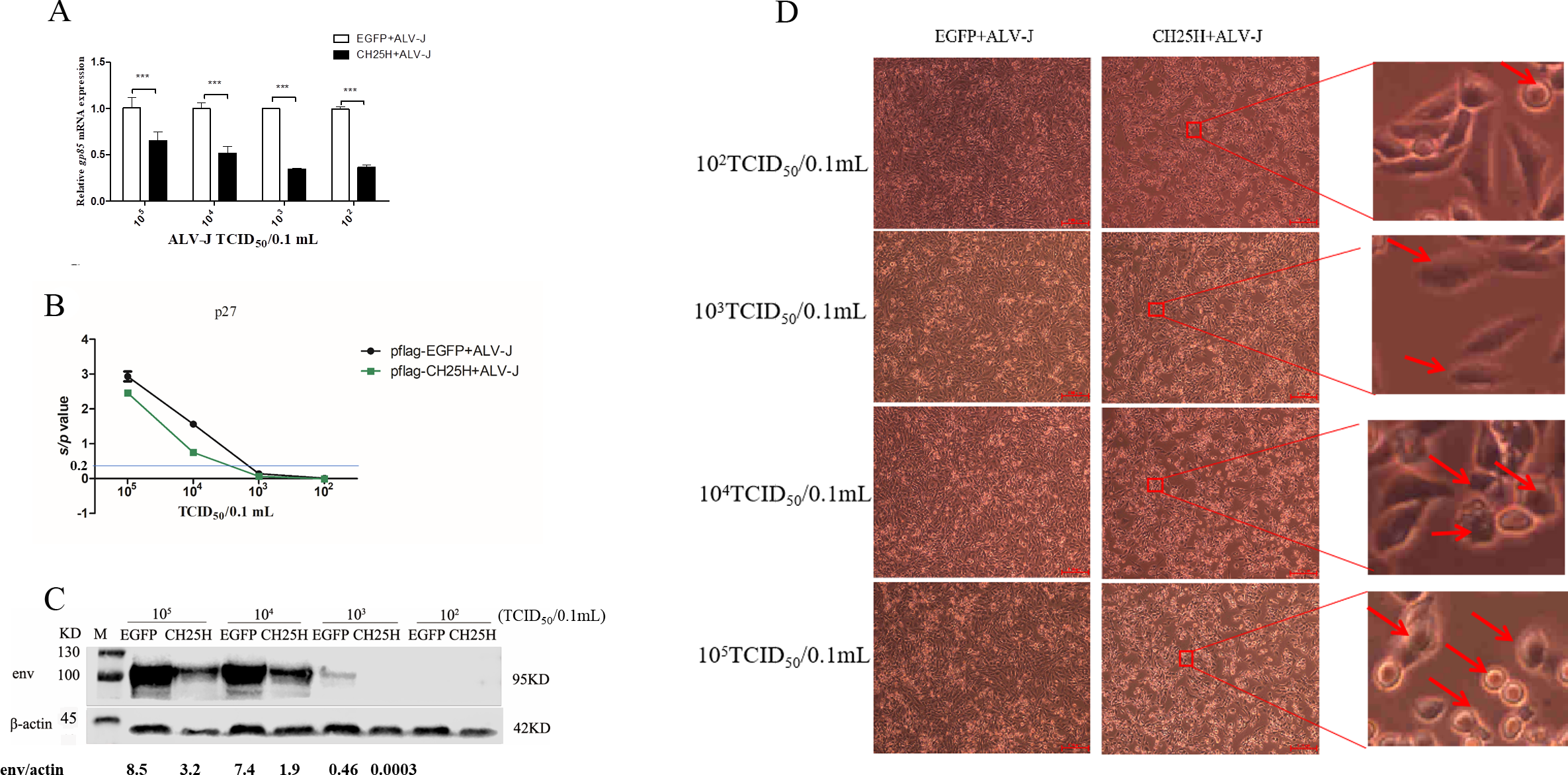

Supplement: Supplementary Figure 1 — CH25H induces host cell death. DF-1 cells were transfected with flag-tagged CH25H for 24 h, subsequently infected with different virus titres of ALV-J for 48 h, respectively (105, 104, 103, 102 TCID50/0.1mL). (A) Relative mRNA levels of ALV-J gp85 were detected by qRT-PCR. (B) The ALV-J p27 protein was detected by ELISA at 24 hpi and 48 hpi. (C) The levels of ALV-J envelope (env) protein were assessed by Western blotting using the specific antibody JE9. (D) The growth of cells were observed under microscope. M represents standard protein markers, CH25H represents the overexpression of Chicken CH25H in DF-1 cells, EGFP represents the control plasmid. Data are shown as the means ± SEM of at least two independent experiments. P values were calculated using unpaired Student’ t-test. Differences with P-values < 0.05 were considered statistically significant differences, and P-values < 0.01 were considered highly statistically significant differences. *** P < 0.001. [file Image_1.tif]

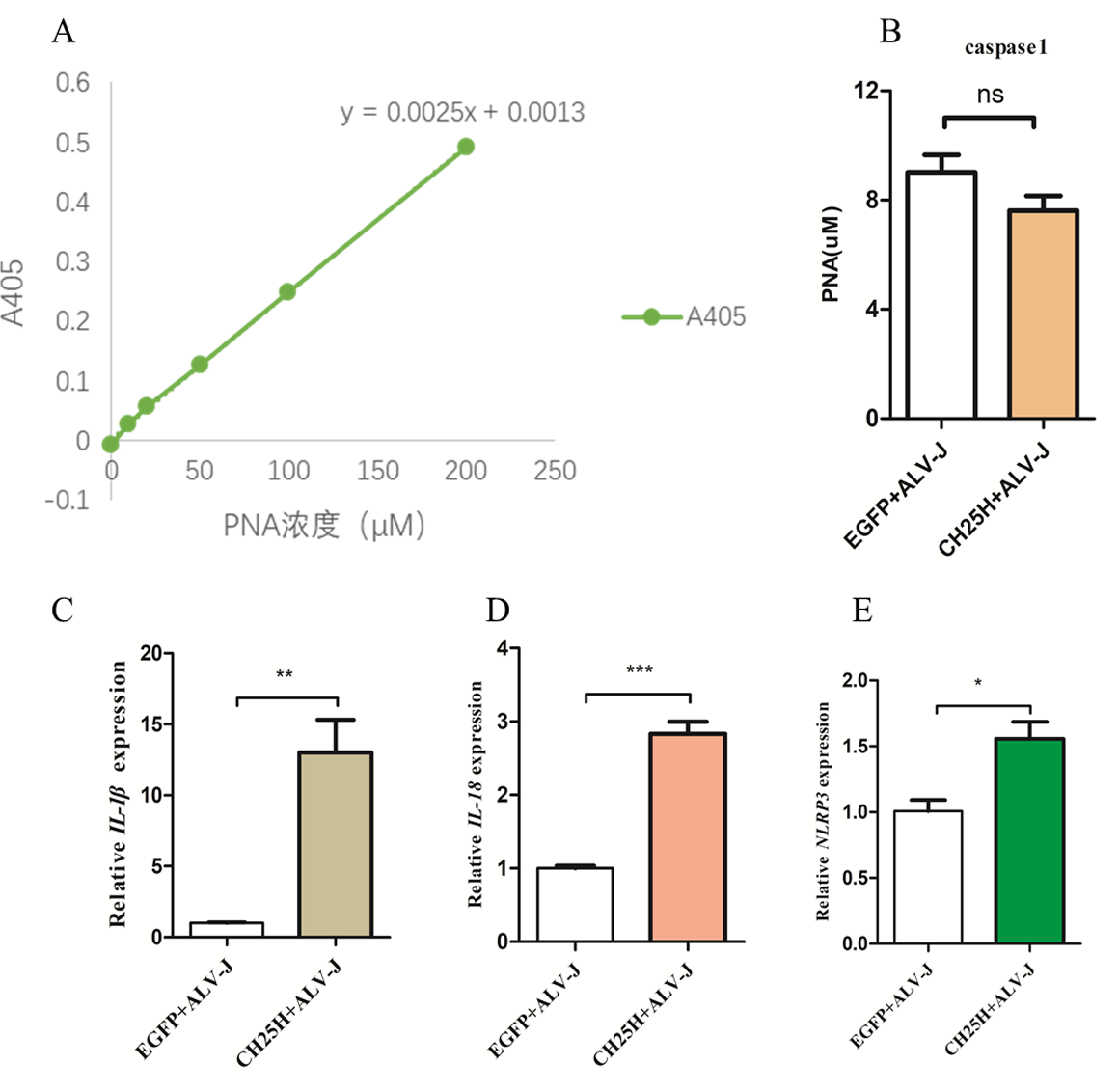

Supplement: Supplementary Figure 2 — CH25H did not induce host cells pyroptosis. DF-1 cells were transfected with flag-tagged CH25H for 24 h, subsequently infected with ALV-J for 48 h, respectively (105 TCID50/0.1mL). (A) A standard curves of pNA. (B) Caspase-1 activity was assayed using a caspase-1 activity assay kit. (C) Relative mRNA levels of IL-1β, IL-18 and, NLRP3 were detected by qRT-PCR. [file Image_2.tif]

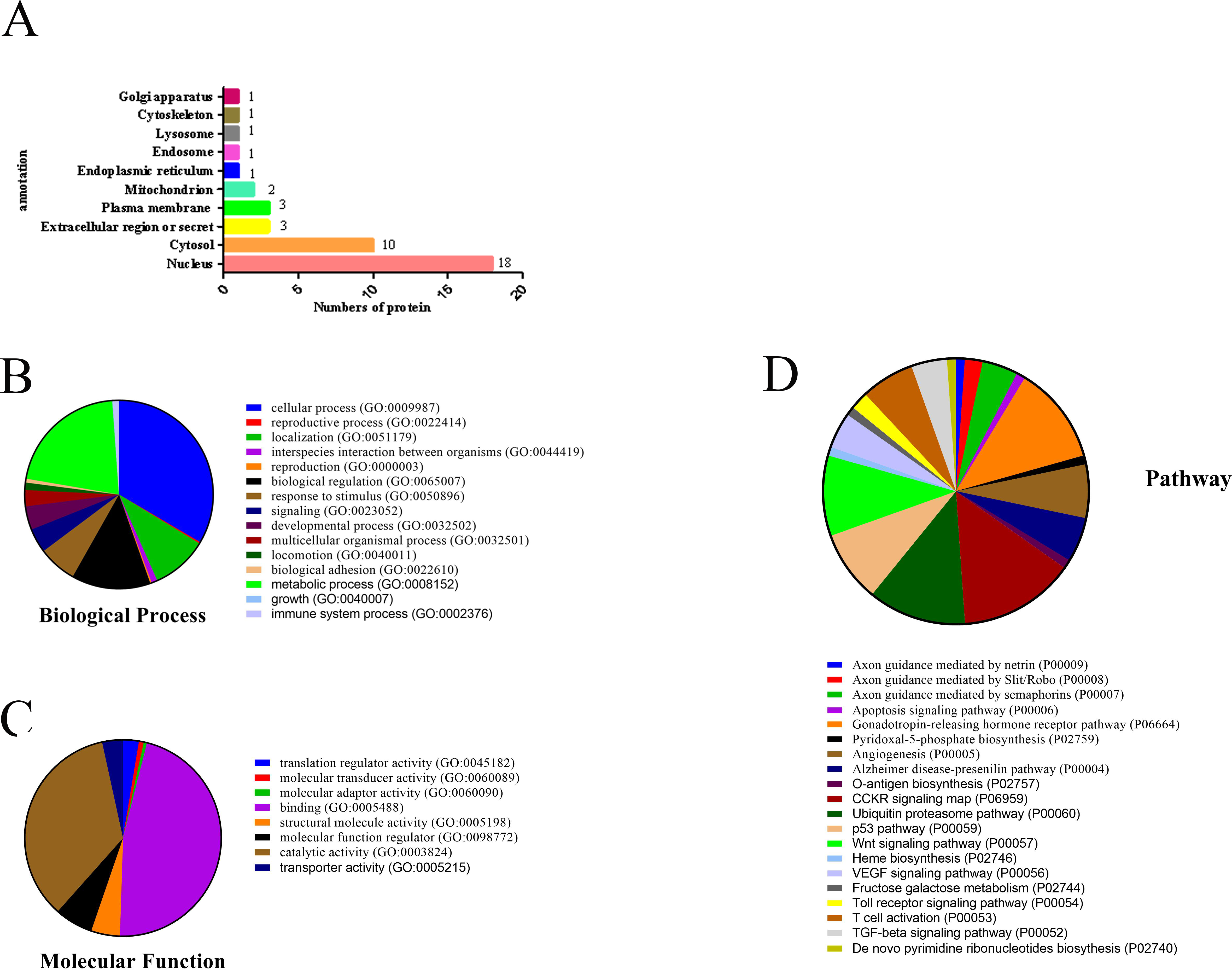

Supplement: Supplementary Figure 3 — Bioinformatics analysis of candidate CH25H-interacting proteins. (A) Subcellular localization of candidate CH25H-interacting proteins as annotated by theUniProt database. (B) Biological process analysis of candidate CH25H-interacting proteins. (C) Molecular funtion of candidate CH25H-interacting proteins. (D) Pathway analysis of candidate CH25H-interacting proteins. [file Image_3.tif]

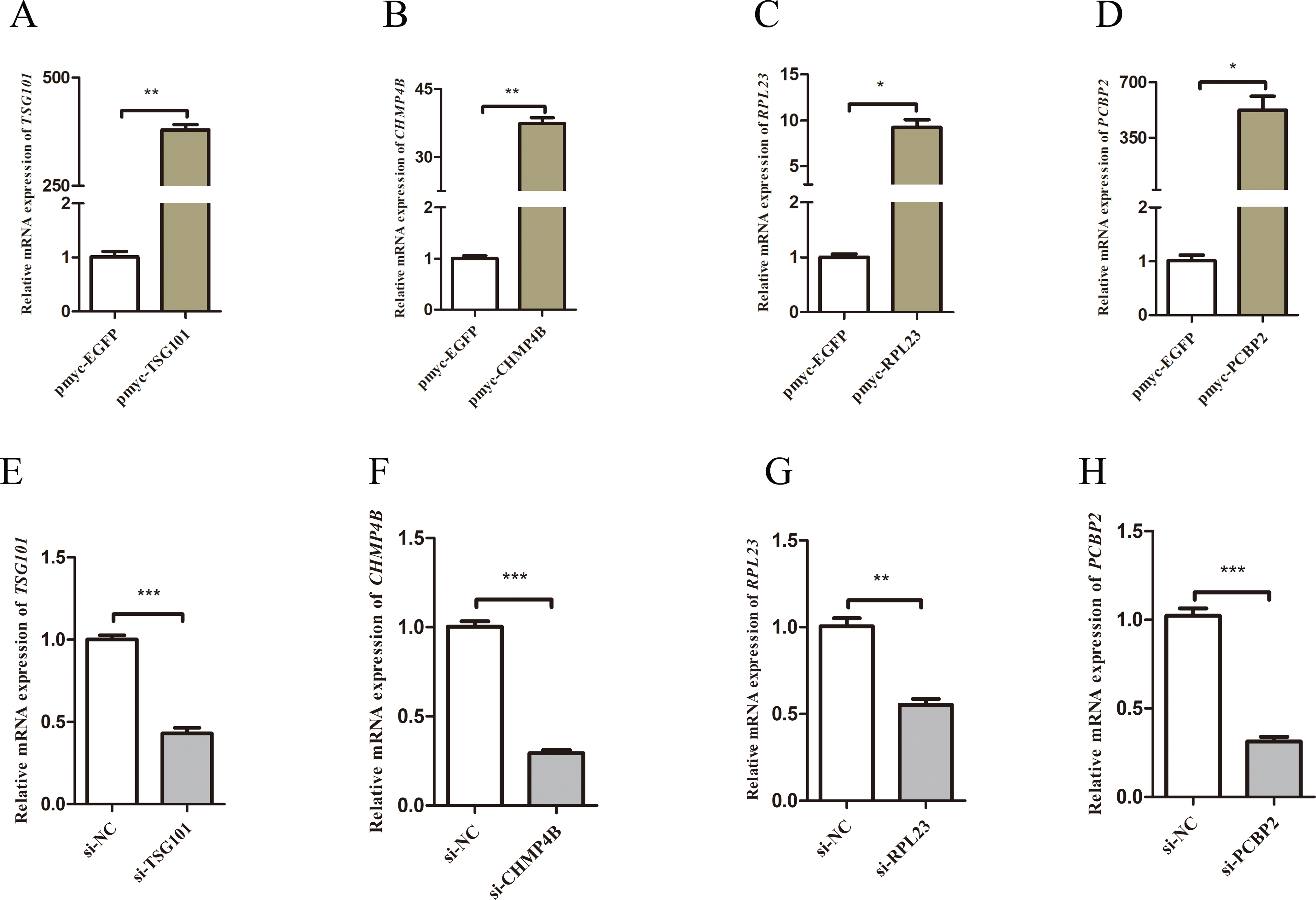

Supplement: Supplementary Figure 4 — The efficiency of overexpression and interference. TSG101 (A), CHMP4B (B), RPL23 (C), and PCBP2 (D) were overexpressed in DF-1cells for 24 h and subsequently infected with ALV-J (105 TCID50/0.1mL), respectively. The efficiency of overexpression was examined by qRT-PCR. Similarly, specific siRNAs were used to silence TSG101 (H), CHMP4B (I), RPL23 (J), and PCBP2 (K) DF-1cells for 24 h and subsequently infected with ALV-J (105 TCID50/0.1mL), respectively. The efficiency of interference was examined by qRT-PCR. [file Image_4.tif]
